# Prey Cue Preferences Among Northern Cottonmouths (*Agkistrodon piscivorus*) Acclimated to Different Year-Long Diets: Genetics or Experienced-Based Plasticity?

**DOI:** 10.1093/iob/obae040

**Published:** 2024-11-08

**Authors:** William I Lutterschmidt, Zander E Perelman, Eric D Roth, J M Weidler

**Affiliations:** Department of Biological Sciences, Sam Houston State University, Huntsville, TX 77340, USA; Illinois Natural History Survey, Prairie Research Institute, University of Illinois Urbana-Champaign, Champaign, IL 61820, USA; Department of Psychology and Brain Sciences, University of Delaware, Newark, DE 19716, USA; South Dakota Game, Fish and Parks, Sioux Falls, SD 57106, USA

## Abstract

Chemoreception and recognition of specific prey are important sensory modalities for optimizing foraging success in snakes. Field observations suggest that cottonmouths are generalists, despite the specific epithet of the species (*piscivorus*) suggesting a fish prey preference. Because chemo-recognition of specific prey may reveal interesting evolutionary context for foraging strategy and if prey preference is either genetically or environmentally controlled, we investigated the prey cue preference of three experimental groups of *Agkistrodon piscivorus* (Northern Cottonmouths) with different diet histories. Two groups of captive snakes were acclimated to year-long diets of either fish (*n* = 11) or mice (*n* = 9) and a third group of recently wild-caught individuals served as a field diet group (*n* = 16). We investigated possible differences among diet history (fish, mouse, and field) and prey cue preference (control, fish, and mouse) and present results showing a significant difference among diet history with field snakes having significantly lower tongue-flick response. We also found a significant difference among prey cues, snakes within all diet histories showed a lower tongue-flick response to only the control scent cue. Both captive and field snakes showed no prey cue preference for either fish or mice. Because captive snakes did not show increased prey cue preference to their respective diet history, prey preference may be under genetic influence and not experience-based. Additionally, the lack of prey preference for fish or mice in the recently captured snakes in the field-diet group provides supporting evidence that *A. piscivorus* are generalists and opportunistic predators.

## Introduction

Chemoreception and recognition of specific prey are important sensory modalities for optimizing foraging success (Schwenk 1995; Cooper 2008). In snakes, the ability to differentiate between chemical cues of prey species (Cooper et al. 1990; Saviola et al. 2012; Saviola and Mackessy 2017) also aids in foraging site selection (Reinert et al. 1984; Roth et al. 1999; Clark 2004a). However, the mechanisms for how such chemo-recognition is acquired has been questioned (e.g., Holding et al. 2016) and may provide important evolutionary context into the natural history of foraging strategies. Prey preference may be influenced by genetic heredity (e.g., Mushinsky and Lotz 1980; Arnold 1981; Waters and Burghardt 2005; Holding et al. 2016; Emerson and Johnson 2023), ontogeny (Saviola et al. 2012), and experience-based plasticity (Loop 1970; Burghardt and Krause 1999; Burghardt et al. 2000; Clark 2004b; Waters and Burghardt 2013). By investigating the mechanisms of chemo-recognition and prey preference, we may gain a better understanding of how selection may favor different foraging strategies and successful acquisition of prey.

Most studies examining chemo-recognition of prey have focused on taxa with specific prey preferences and active foraging modes (e.g., Mushinsky and Lotz 1980; Burghardt et al. 2000; Waters and Burghardt 2005; Cooper and Secor 2007). In this study, we used a foraging generalist as an alternative experimental model to investigate if specific year-long experienced-based diets could influence prey preference. The first taxonomic descriptions refer to the Cottonmouth (*Agkistrodon piscivorus*, Lacépède 1789) as “Le Piscivore,” suggesting a prey preference for fishes, and although the bulk of its prey taken in nature consists of fishes and frogs (Gloyd and Conant 1990), observations suggest that *A. piscivorus* are generalists and opportunistic predators more appropriately referred to as omnicarnivorous (Ditmars 1912). The diet of *A. piscivorus* encompasses a wide diversity of prey including invertebrates, fishes, amphibians, reptiles, birds, mammals, and even carrion (e.g., Conant 1929; Gloyd and Conant 1990; Roth et al. 2003; Vincent et al. 2004; Schalk et al. 2018; Hofmann et al. 2023). Additionally, *A. piscivorus* engage in different foraging modes including sit-and-wait foraging (Eskew et al. 2009), active foraging (Lillywhite and McCleary 2008; Eskew et al. 2009), and scavenging (Devault and Krochmal 2002; Lillywhite and Brischoux 2012).

We used the study presented by Holding et al. (2016) and the tongue-flick response (Burghardt 1967) as ideal models to investigate a similar, but different question regarding genetically or environmentally controlled prey scent cue preference in *A. piscivorus*. Because *A. piscivorus* are prey generalists, we would expect a genetically influenced non-specific preference among chemical prey cues. However, under an environmentally controlled experience-based model, where foraging success affects future prey preference (Clark 2004b), we would expect *A. piscivorus* to show more plasticity in prey-cue preference respective to their year-long experienced-based diet of either fish or mice.

## Materials and methods

### Experimental subjects, captive care, and diet

Adult *A. piscivorus* used in this study were collected in July 2016 from Harmon Creek located in Walker County, Texas. Snakes (*n* = 24) were randomly selected and placed in either a fish (golden shiners, *Notemigonus crysoleucas*) or mice (CD-1^®^ IGS Laboratory mouse, *Mus musculus*) diet group as part of the study by Weidler and Lutterschmidt (2021) investigating diet-influenced cutaneous water loss. A fish versus mammal prey diet also reflects the main aquatic (Malloy 1971) and terrestrial (Lutterschmidt et al. 1996, 2021; Schalk et al. 2018) prey items taken from their dichotomous use of aquatic and terrestrial microhabitats while foraging.

Each snake was housed separately in plastic cages (38 × 26 × 22 cm) with aspen bedding (Harlan Teklad, Madison, Wisconsin) and water provided ad libitum. Snakes were kept in a laboratory and acclimatized to temperature (25 ± 2°C), relative humidity (50 ± 3%), and photoperiod (12L:12D cycle) with the photophase centered on 1200 h. Beginning September 2016, snakes were fed weekly and offered either fish or mice equal to 20% of their M_b_ (Lutterschmidt and Rayburn 1993; Byars et al. 2010; Sparkman et al. 2010). We measured each snake's initial snout-vent-length (SVL) to the nearest 0.1 cm (mean = 51.07, SE = 1.167, *n* = 24) and body mass (M_b_) to the nearest gram (mean = 193.9, SE = 11.98, *n* = 24) and M_b_ and SVL were measured monthly (September 2016 to July 2017) for changes in M_b_ and SVL resulting from growth.

Of the 24 snakes within this captive colony, we used individuals that had completed their shedding sequence (Weidler and Lutterschmidt 2021) from the fish (*n* = 11) and the mouse (*n* = 9) diet groups. Prior to experiments of prey cue preference beginning July 22, 2017, all captive snakes received their last feeding in the first week of July to control hunger (Holding et al. 2016). In addition to captive snakes, recently captured snakes (*n* = 16) collected from the field in early July were included as a field-diet group. These field snakes were assumed to have recently fed under natural conditions and were not offered food to avoid possible influences on prey preference, and thus, had similar hunger levels as captive snakes.

### Quantifying tongue-flick response to scent cues

The functional morphology of the vomeronasal organ and tongue-flicking to perceive chemical cues in the environment (Halpern 1992) is well established and serves as an ideal behavioral model for investigating chemoreception and scent cue preference (e.g., Burghardt 1967, 1970; Fuchs and Burghardt 1971; Cooper and Burghardt 1990; Cooper and Secor 2007; Holding et al. 2016; Goetz et al. 2018). Snakes of the genus *Agkistrodon* tongue-flick in response to prey chemical cues both under laboratory (Chiszar et al. 1979) and field (Young et al. 2008) manipulations, and it has been suggested that *Agkistrodon* species rely more on chemosensory information while foraging than other North American pitvipers (Lillywhite et al. 2002).

We prepared fish and mouse scent extracts following procedures similar to Clark (2004a) by suspending prey items in a 350 mL beaker of distilled water for 3 h. The solution was then transferred to a volumetric flask, sealed, and refrigerated at 4°C until use over the next 3 days. Pure distilled water served as the control scent cue.

Experiments of scent cue preference were conducted on 22, 23, and 24 July 2017 beginning at 2100 h and ending by 0100 h to coincide with documented activity patterns in this population (Lutterschmidt et al. 2022). The presentation order of scent cues (*n* = 3) was randomly determined for each snake with snakes being tested only once per night. The scent cue sequence was completed with testing over three-consecutive trial nights. The testing order of snakes (*n* = 36) each night was also randomized to avoid testing of the same snake at the same time.

An hour prior to experimental trials, we allowed 100 mL of each of our three stock solutions within a labeled screw-top sample jar to reach room temperature (25°C). Solutions were capped and kept from the proximity of our testing cage. The sides of the testing cage (38 × 26 × 22 cm) were opaque to eliminate visual cues and the top was one-way glass to allow for experimenter observation of the snake and tongue-flicks. Each snake was placed in the testing cage and allowed to habituate for 5 min prior to presenting scent cues. Snakes usually assumed a relaxed coiled body position within the 5 min habituation period.

A Pur-Wraps^®^ 6-inch sterile cotton tipped applicator swab was dipped into the scent extract and the opposite end attached to a 50 cm long × 0.5 cm wide wooded rod to extend the applicator. A small round access port (5 cm diameter) on top of the testing cage was opened and the scent introduced by holding the cotton swab approximately 5 cm in front of the snake's head and held motionless. An initial tongue-flick began the trial with two observers recording the number of tongue-flicks over a 60 s period. Other behaviors such as striking, biting, and bite latency (e.g., Holding et al. 2016) were also recorded.

### Data analysis

Tongue-flick counts from the two observers were averaged. A tongue-flick attack score (Cooper and Burghardt 1990; Holding et al. 2016) was not used in our analysis due to the infrequency of striking and biting (i.e., 4 observations out of 108 trials). Tongue-flick counts were tested for assumptions of normality (Shapiro–Wilks) and equal variance (Levene's) and we used a Model-I, 3 × 3 factorial, repeated measures two-way ANOVA without replication with diet history (fish, mouse, and field) and scent cue (control, fish, and mouse) as the two fixed factors. Scent cue served as the repeated measure within random subjects (i.e., individual snakes) accounting for possible individual variation in tongue-flick response. Significant differences among diet histories and scent cue were investigated using the Holm-Sidak multiple comparisons procedure for all pairwise comparisons including the control scent cue of distilled water. We used SigmaPlot^®^ 11.0 for all statistical analyses and graphing.

## Results

All snakes demonstrated a tongue-flick response in each of the scent cue trials (Table 1). Differences in observed tongue-flicks between experimenters occurred in 18 of the 108 trials with count differences of 1 (*n* = 15), 2 (*n* = 2), and 3 (*n* = 1). For these trials, tongue-flick counts were averaged. Observations of striking and biting (*n* = 4 of 108 trials; 3.7%) were infrequent and not used to generate tongue-flick attack scores or used in the analysis.

**Table 1:** Tongue-flick (TF) response (TF count per minute) of *A. piscivorus* to a 60 s presentation to control, fish, and mouse sent cues on a cotton swab.

Diet-history group	Scent cue	Sample (*n*)	TF response	Trials with strike or bite
Fish diet	Control	11	12.73 ± 3.037	0
	Fish	11	25.73 ± 3.096	1
	Mouse	11	21.41 ± 4.836	3
Mouse diet	Control	9	14.50 ± 2.890	0
	Fish	9	20.28 ± 2.859	0
	Mouse	9	22.83 ± 5.057	0
Field diet	Control	16	5.66 ± 1.562	0
	Fish	16	15.50 ± 2.101	0
	Mouse	16	10.16 ± 2.521	0

All trials (*n* = 36) had at least one TF response. The mean TF response is shown with standard error (±SE).

**Fig. 1:**
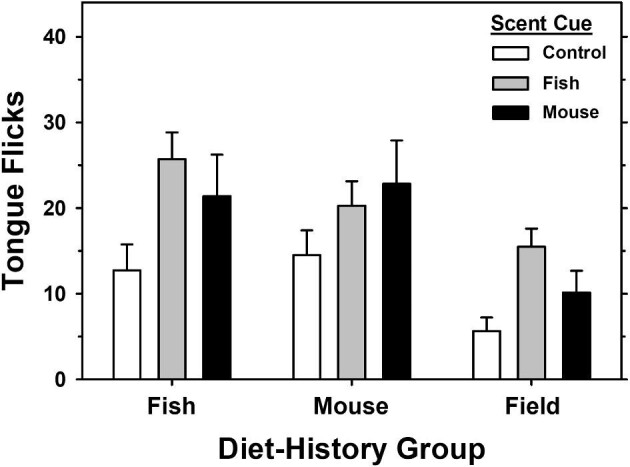
Mean (±SE) tongue-flick response (frequency of tongue flicks in 60 s interval) for both diet history and prey-scent cue stimuli.

The repeated measures two-way ANOVA (Table 2) indicated statistically significant differences among diet-history groups (*F*_2,66_ = 7.06, *P* = 0.003) and scent cue stimuli (*F*_2,66_ = 9.94, *P* < 0.001) with no interaction between factors (*F*_4,66_ = 0.78, *P* = 0.544). Holm-Sidak multiple comparison procedures indicated that captive snakes in both the fish and mouse diet-history groups did not differ in tongue-flick response (*t* = 0.23, *P* = 0.820). However, the recently wild-caught snakes in the field-diet history group differed significantly with a lower tongue-flick response than snakes in the fish (*t* = 3.33, *P* = 0.006) and mouse (*t* = 2.88, *P* = 0.014) diet groups (Fig. 1).

**Table 2:** Results of the 3 × 3 factorial two-way repeated measures ANOVA with diet history and scent cue as factors.

Source of variation	df	SS	MS	*F*	*P*
Diet history	2	2255.23	1127.61	7.06	0.003
Snakes	33	5266.62	159.60		
Prey-scent cue	2	1679.26	839.63	9.94	0.001
Interaction (diet × scent)	4	262.52	65.63	0.78	0.544
Residual	66	5575.42	84.48		
Total	107	15,166.85	141.75		

Individual snakes serve as the repeated measure to control for individual variation in tongue-flick (TF) response among individuals. Assumptions of normality (Shapiro–Wilks, *P* = 0.467) and equal variance (Levene's *P* = 0.099) for analyses were tested and passed.

As expected, snakes across all diet-history groups showed a significantly lower tongue-flick response to the control scent cue of distilled water (*F*_2,66_ = 9.94, *P* < 0.001) compared to the fish (*t* = 4.28, *P* < 0.001) and mouse (*t* = 3.22, *P* = 0.004) scent cues. However, snakes across diet groups did not show a significant preference for either the fish or mouse scent cues (*t* = 1.06, *P* = 0.292).

## Discussion

Year-long acclimation to either a fish or mouse diet did not influence prey cue preference in *A. piscivorus*, supporting our prediction that prey preference is not experience-based but rather under genetic influence. Such a genetic non-specific prey preference would favor a more opportunistic foraging strategy by *A. piscivorus*, and the lack of prey preference for fish or mouse scent cues in both captive and recently wild-caught snakes in the field-diet group provides supporting evidence that *A. piscivorus* are generalists and opportunistic predators. It is possible that previous field-foraging experience influenced tongue-flick results for *A. piscivorus* in the fish and mouse diet groups, as they were wild-caught adults at the onset of diet-manipulation. However, this explanation would suggest *A. piscivorus* prey preference is experience-based. Thus, we would subsequently expect that year-long feeding experience from a manipulated diet would influence trials results (Clark 2004b), which was not the case here as snakes displayed no clear preference for either prey cue type.

Holding et al. (2016) found that *Sistrurus miliarius* are not influenced by experience-based diets, but in contrast, show a specific prey preference of lizards despite eating a variety of prey (Gibbs and Mackessy 2009). Emerson and Johnson (2023) found that naïve neonate *Crotalus ornatus* showed a preference for native prey types, despite a total lack of previous exposure to such chemical cues. In all three experimental models (Holding et al. 2016; Emerson and Johnson 2023; the present study), results provide evidence for genetic influence of prey preference in *S. miliarius, C. ornatus*, and the absence of prey preference in *A. piscivorus*, respectively. These results have evolutionary significance and reflect each species’ evolutionary life history strategies for foraging and feeding. Because chemoreception and prey recognition may be under divergent selection among areas with varying availability of specific prey species (Holding et al. 2016), genetically determined dietary plasticity in *A. piscivorus* may be an important evolutionary trait in foraging success by a semi-aquatic snake using a diversity of habitats (Lillywhite et al. 2015).

Although *A. piscivorus* may forage mostly on fishes and frogs (Gloyd and Conant 1990), our results suggest that this observation is most likely due to prey availability, not prey preference. Because *A. piscivorus* are semi-aquatic, the complexity, diversity, and integration of habitat use between aquatic and terrestrial habitat types and their ecotones supports opportunistic and omnicarnivorous (Ditmars 1912) foraging strategies that might regularly introduce novel prey (e.g., Lutterschmidt et al. 1996, 2021). Additionally, a non-specific prey preference and an opportunistic foraging strategy may also be advantageous in avoiding competition (Lutterschmidt et al. 2007) when *A. piscivorus* populations (as studied here) are sympatric with *A. contortrix*, a closely related congener.

Population-level comparisons for the absence or presence of prey preference within *A. piscivorus* would be an interesting extension of the presented research. Do *A. piscivorus*, which are not largely sympatric with *A. contortrix*, show a greater degree of preference for specific prey? Is there population-level geographic variation and plasticity in the absence of prey preference based upon degrees of aquatic and terrestrial habitat integration or the degree of overlap with closely related sympatric species? Future research may also investigate potential preference for other prey-scent cues not tested here (e.g., frogs, snakes, lizards). This experimental model for a genetically determined lack of prey preference offers a unique system for investigating how this behavioral phenotype evolved with specific natural histories and foraging strategies (Arnold 1981; Gomulkiewicz et al. 2007). The mechanisms controlling the absence or presence of prey-cue preference may provide insight for how and why different foraging strategies have evolved.

## Data Availability

All data underlying this article is provided within the manuscript.
